# Cartilage stem/progenitor cells are activated in osteoarthritis via interleukin-1β/nerve growth factor signaling

**DOI:** 10.1186/s13075-015-0840-x

**Published:** 2015-11-17

**Authors:** Yangzi Jiang, Changchang Hu, Shuting Yu, Junwei Yan, Hsuan Peng, Hong Wei Ouyang, Rocky S. Tuan

**Affiliations:** Center for Cellular and Molecular Engineering, Department of Orthopaedic Surgery, University of Pittsburgh School of Medicine, 450 Technology Drive, Pittsburgh, PA 15219-3143 USA; Dr. Li Dak Sum & Yip Yio Chin Center for Stem Cell and Regenerative Medicine, Zhejiang University, School of Medicine, Hangzhou, Zhejiang 310058 China; Tsinghua University School of Medicine, Beijing, 100084 China; Current address: Vascular Surgery, The Affiliated Hospital of Qingdao University, Qingdao, 266071 China; Current address: Berea College, Berea, KY 40403 USA

**Keywords:** Cartilage stem/progenitor cells, Stem cells, Interleukin-1β, Nerve growth factor, Osteoarthritis, Signaling

## Abstract

**Introduction:**

Interleukin-1β (IL-1β) and nerve growth factor (NGF) are key regulators in the pathogenesis of inflammatory arthritis; specifically, IL-1β is involved in tissue degeneration and NGF is involved in joint pain. However, the cellular and molecular interactions between IL-1β and NGF in articular cartilage are not known. Cartilage stem/progenitor cells (CSPCs) have recently been identified in osteoarthritic (OA) cartilage on the basis of their migratory properties. Here we hypothesize that IL-1β/NGF signaling is involved in OA cartilage degeneration by targeting CSPCs.

**Method:**

NGF and NGF receptor (NGFR: TrkA and p75NTR) expression in healthy and OA human articular cartilage and isolated chondrocytes was determined by immunostaining, qRT-PCR, flow cytometry and western blot. Articular cartilage derived stem/progenitor cells were collected and identified by stem/progenitor cell characteristics. 3D-cultured CSPC pellets and cartilage explants were treated with NGF and NGF neutralizing antibody, and extracellular matrix changes were examined by sulfated glycosaminoglycan (GAG) release and MMP expression and activity.

**Results:**

Expression of NGF, TrkA and p75NTR was found to be elevated in human OA cartilage. Cellular changes upon IL-1β and/or NGF treatment were then examined. NGF mRNA and NGFR proteins levels were upregulated in cultured chondrocytes exposed to IL-1β. NGF was chemotactic for cells isolated from OA cartilage. Cells isolated on the basis of their chemotactic migration towards NGF demonstrated stem/progenitor cell characteristics, including colony-forming ability, multi-lineage differentiation potential, and stem cell surface markers. The effects of NGF perturbation in cartilage explants and 3D-cultured CSPCs were next analyzed. NGF treatment resulted in extracellular matrix catabolism indicated by increased sGAG release and MMP expression and activity; conversely, treatment with NGF neutralizing antibody inhibited increased MMP levels, and enhanced tissue inhibitor of matrix metalloprotease-1 (TIMP1) expression in OA cartilage explants. NGF blockade with neutralizing antibody also affected cartilage matrix remodeling in 3D-CSPC pellet cultures.

**Conclusion:**

Our results strongly suggest that NGF signaling is a contributing factor in articular cartilage degeneration in OA, which likely targets a specific subpopulation of progenitor cells, the CSPCs, affecting their migratory and matrix remodeling activities. These findings provide novel cellular/signaling therapeutic targets in osteoarthritic cartilage.

**Electronic supplementary material:**

The online version of this article (doi:10.1186/s13075-015-0840-x) contains supplementary material, which is available to authorized users.

## Introduction

The degenerative joint disease osteoarthritis (OA) is a major cause of mobility loss, and represents the most prevalent form of musculoskeletal disease worldwide [1, 2]. The Centers for Disease Control and Prevention (CDC) reports that 52.5 million US adults had arthritis in 2010–2012, and estimated up to 67 million by the year 2030 [3]. Current clinical OA management is mainly concerned with symptom reduction (e.g., pain, swelling, stiffness) [4], with oral nonsteroidal anti-inflammatory drugs (NSAIDs) being the most commonly used pharmacological treatment at mid-stage of the disease, and arthroplasty, an irreversible procedure, as the final solution to maintain joint function. The pathogenesis of OA is incompletely understood. Better understanding of the mechanisms of OA progress is thus critical for development of an intervention method.

The primary target tissue of OA is the articular cartilage, a physiologically nonself-renewing, avascular tissue that functions as the load-bearing joint surface. Cartilage tissue is composed of over 90 % extracellular matrix (ECM) and less than 10 % chondrocytes in total volume. Collagens and proteoglycans (PGs) present in the ECM are the major macromolecules directly responsible for the load-bearing properties of the hyaline articular cartilage [5]. Joint cartilage destruction is triggered by a number of events, including changes in the mechanical environment, biochemical alterations of the ECM, and biological responses of chondrocytes, the subchondral bone, and the synovium to inflammation.

Proinflammatory cytokines, such as interleukin (IL)-1β, are key regulators in the pathogenesis of inflammatory arthritis, including tissue degeneration. IL-1β induces production of major extracellular proteolytic enzymes in chondrocytes, such as matrix metalloproteinases (MMPs) and a disintegrin-like and metalloproteinase with thrombospondin motifs (ADAMTS) [6, 7]. Wheaton et al. [8] reported that intraarticular injection of recombinant porcine IL-1β into the pig knee joint resulted in cartilage loss. Lai et al. [9] reported that induction of IL-1β in healthy mouse joints could cause joint structure changes, dysfunction, and pain. IL-1β could also lead chondrocytes to express more proinflammatory cytokines such as tumor necrosis factor alpha (TNFα), IL-8, complement factors, and prostaglandin E_2_ [7, 10–12]. IL-1β is an important therapeutic target of OA, and drugs that block IL-1β action have been developed and in use for decades [10].

Nerve growth factor (NGF), an important signaling molecule involved in joint pain, is a key regulator of rheumatoid arthritis pathogenesis, and has recently been implicated in OA pain. NGF and nerve growth factor receptor (NGFR) may act in OA pain through at least two mechanisms: increasing proliferation of sensory neurons [13]; and changing the pain threshold through high-affinity NGFR (TrkA)-mediated sensitization of sensory neurons [14]. A recent clinical trial showed that intravenous injection of the NGF neutralizing antibody, Tenezumab^®^ (Pfizer, New York, NY, USA) can relieve OA joint pain [15]. These functional effects are likely to occur within synovial tissue [16, 17] and osteochondral junctions [18], as well as in dorsal root ganglia [19–21]. Interestingly, other research findings also suggest NGF involvement in cytokine-mediated OA pathogenesis, with articular cartilage as a target tissue. For example, articular cartilage is immunopositive for NGF, and higher expression of NGF and TrkA [22] is observed in OA [23]. Furthermore, genome-wide association scan results revealed the association of an NGF variant, MCF2L, with OA [24], suggesting that OA may be related to interruption of NGF functional balance. At present, the role of NGF/NGFR in articular cartilage biology and OA pathogenesis is unclear.

Cartilage stem/progenitor cells (CSPCs) have recently been found within human articular cartilage [25–28]. These cells are thought to respond to injury and migrate through the disease-affected tissue zones, and are characterized as stem/progenitor cells in vitro by virtue of their self-renewal ability, differentiation multipotency, and stem cell markers [29, 30]. Expression of several mesenchymal progenitor cell markers, including Stro-1, VCAM (CD166), Notch-1, etc. [31], has been detected in OA but not in normal human articular cartilage, suggesting that OA may activate an endogenous CSPC population within the tissue. The signaling events in cartilage degeneration are unclear. Recent studies showed induction of CSPC homing by injured cartilage conditioned medium, dead cell debris, and chromosomal proteins [30], suggesting soluble cell recruitment signals.

Here we hypothesized that IL-1β/NGF signaling is involved in OA cartilage degeneration by targeting CSPCs. The hypothesis is tested by: characterizing NGF and NGFR expression in OA cartilage; assessing the effect of IL-1β treatment on NGF/NGFR in cartilage explants and in cartilage-derived CSPCs; and examining the effects of NGF perturbation on the cartilage-forming activities of CSPCs in three-dimensional (3D) cultures. Our findings strongly suggest coordinated signaling by IL-1β and NGF in cartilage degeneration via CSPCs, which represent a potential therapeutic target for OA.

## Methods

### Tissue harvest and explant culture

Cartilage explant cultures were taken from macroscopically unaffected areas of the femoral condyle from total knee arthroplasty (Institutional Review Board approval, consent exempted: University of Pittsburgh, PA, USA and University of Washington, Seattle, WA, USA). Adult articular cartilage was obtained from nine females and six males (average age, 60.1 years). Histological slides of normal adult (*n* = 3) and fetal articular cartilage (*n* = 3) were donated by the Department of Pathology and Anatomy, Zhejiang University School of Medicine, Zhejiang, China, and were examined by histological staining and immunohistochemistry.

To study the effects of NGF treatment on tissue morphology, a white, smooth piece of articular cartilage (~1 cm × 1 cm and 3–5 mm in depth) harvested from a nonlesion area of the joint surface was cut orthogonally into ~2 mm sized pieces, and was cultured with varying concentrations of NGF for up to 14 days. At the end of culture, the explants were fixed in 4 % paraformaldehyde and processed for histology. Sections of 14 μm thickness were examined histologically using Safranin-O/Fast Green staining.

To examine the effects of NGF treatment on cartilage matrix degeneration, cartilage explants were cultured with serum-free medium for 12 hours, and dispersed at the same wet weight (0.5–1 g/well, depending on each donor) into a multiwell (12-well or 24-well) plate. The same volume of culture medium with or without NGF and/or anti-NGF antibody was added to each well (0.3 g wet weight tissue/ml culture medium) and subsequently collected at various time intervals up to 2 weeks to assay for release of sulfated glycosaminoglycan (sGAG) and MMPs. The freshly isolated tissue was denoted as the day 0 sample.

The culture medium consisted of Dulbecco’s Modified Eagle’s medium (DMEM; Life Technologies, Thermo Fisher, Grand Island, NY, USA), supplemented with ITS+ and 1 % penicillin–streptomycin (Life Technologies), with 1 ml medium for ~300 mg tissue. Treatments included 10 ng/ml recombinant human NGF-β (Sigma, St. Louis, MO, USA), and/or 10 ng/ml NGF neutralizing antibody (ab16161; Abcam, Cambridge, MA, USA). Medium was removed and collected every 2 days during the 2-week culture period.

### Cell isolation, culture, and characterization

Primary human chondrocytes from distal femoral condyles were isolated by enzymatic digestion. Briefly, the tissue was minced into ~1 mm^3^ pieces and digested for 6 hours at 37 °C with a 0.2 % solution of collagenase type I (Life Technologies). Cells were transferred to monolayer culture in DMEM/Ham’s F12 Medium (1:1; Life Technologies), supplemented with 10 % fetal bovine serum (FBS) and penicillin/streptomycin, and cultured under standard conditions. For the NGF release test, early-passage (before passage 2) chondrocytes were cultured as a high-density monolayer (>95 % confluence) and treated with 100 pg/ml IL-1β for 1 week. The medium was changed on days 1, 4, and 7 and collected for NGF enzyme-linked immunosorbent assay (ELISA; R&D Systems, Minneapolis, MN, USA). RNA was isolated on day 7.

Based on the migratory ability of CSPCs, we collected the NGF responsive CSPCs from a Transwell system (see Cell migration assay). The NGF-attracted cells were trypsinized from the bottom of the Transwell insert and cultured in growth medium. Stem cell characteristics were tested as described in the following by flow cytometric analysis, and on the basis of multilineage differentiation and colony-forming ability. To test the effects of NGF on CSPCs, migratory cells were cultured as a high-density monolayer (>95 % confluence). After treatment in serum-free medium for 12 hours, cells were treated with 10 ng/ml NGF or 100 pg/ml IL-1β for 48 hours and RNA was extracted for quantitative RT-PCR analysis of gene expression.

To investigate the effect of NGF on cell proliferation, cells were seeded in 96-well plates at a density of 3000 cells/cm^2^ in a medium containing 2 % FBS. After attachment, cells were treated with 10 or 100 ng/ml NGF, 10 ng/ml NGF neutralizing antibody, or the combination of 10 ng/ml NGF and antibody for 1, 3, 5, and 7 days. Cell proliferation was tested using the CCK-8 Kit (DOJINDO, Rockville, MD, USA).

### Immunostaining

For immunostaining, tissue specimens were fixed in 4 % paraformaldehyde and processed as cryosections, while cells were transferred to 24-well plates and cultured for 16 hours, after which they were fixed with 4 % paraformaldehyde. The samples were incubated with mouse monoclonal antibodies (human CD271; BD Pharmingen, San Diego, CA, USA), followed by fluorescein isothiocyanate (FITC)-conjugated, secondary rabbit anti-mouse IgG (Life Technologies). Cell nuclei were counterstained with 4′,6-diamidino-2-phenylindole (DAPI), or with a Histostain Kit according to the manufacturer’s protocol (Life Technologies). Immunostained sections and cells were examined by phase-contrast and epifluorescence microscopy using an Olympus i X71 microscope (Olympus, Tokyo, Japan).

### Flow cytometric analysis

Antibodies (from Pharmingen/BD Biosciences, San Diego, CA, USA; unless specified otherwise) used for flow cytometry included: FITC-conjugated specific antibodies to human CD34 (clone 581, IgG1,κ), CD90 (clone 5E10, IgG1,κ), CD45 (clone 30-F11, IgG2a,κ; eBioscience, San Diego, CA, USA); phycoerythrin (PE)-conjugated mouse-specific antibody to human CD44 (clone 515, IgG1,κ); and nonconjugated mouse-specific antibody to human CD105 and CD271, detected with FITC-conjugated rabbit anti-mouse IgG (Life Technologies). PE-conjugated or FITC-conjugated isotype-matched IgGs were used as controls. Cells (5 × 10^5^ cells/ml) were incubated with primary antibodies for 30 minutes at 4 °C. After washing, the cells were fixed with 1 % paraformaldehyde and analyzed using a Beckman-Coulter FC 500 MCL/MPL flow cytometer (Brea, CA, USA).

### Cell migration assay

The migratory activity of chondrocytes isolated from healthy and OA articular cartilage at different passages was assessed as follows. Fifty thousand cells were seeded in the upper insert of a Transwell plate (Costar 3422, Corning, NY, USA) and cultured in serum-free DMEM. Serum-free medium, containing NGF (10 ng/ml) and/or NGF neutralizing antibody (10 ng/ml), was added to the lower chamber. After 24-hour incubation, the inserts were removed and the upper surface of the filter was scraped free of cells and debris. After staining with DAPI, the number of migrated cells detected on the underside of the Transwell filter was counted in five randomly selected fields (100× magnification), and the results per microscopic field are expressed as mean ± standard deviation (SD). Data presented are representative of three independent experiments.

### Multilineage differentiation potential

The differentiation potential of CSPCs was tested on the basis of induced osteogenesis, adipogenesis, and chondrogenesis. For osteogenesis, cells were plated at low density (1 × 10^3^ cells/cm^2^) in a 24-well plate and cultured with 10 mM β-glycerolphosphate (Sigma), 0.1 μM dexamethasone (Sigma), and 50 μg/ml ascorbate (Sigma) in DMEM–high glucose medium containing 10 % FBS and 1 % penicillin–streptomycin for 2 weeks. Osteogenesis was assessed by alkaline phosphatase (ALP) histochemical staining (ALP kit; Beyotime Biotechnology, Shanghai, China). For adipogenesis, confluence cultures were treated with 1 mM dexamethasone, 10 μg/ml insulin, and 0.5 mM isobutylxanthine (all Sigma) in DMEM–high glucose medium  containing 10 % FBS and 1 % penicillin–streptomycin for 2 weeks. Intracellular accumulation of lipid droplets was detected by Oil Red O (Sigma) staining. For chondrogenesis, high-density pellets of 2 × 10^5^ cells were formed by centrifugation in U-shaped bottom 96-well plates and cultured in 10 ng/ml TGF-β3 (PeproTech, Rocky Hill, NJ, USA), 50 μg/ml ascorbic acid (Sigma), 40 μg/ml proline, and 0.1 μM dexamethasone in DMEM–high glucose medium containing ITS and 1 % penicillin–streptomycin. The medium was changed every 2–3 days, and cell pellets were observed at intervals of 14 and 21 days. To examine the influence of NGF, some pellets were treated with 10 ng/ml human recombinant NGF-β, 10 ng/ml NGF neutralizing antibody, or 10 ng/ml NGF neutralizing antibody matched isotype (mouse IgG_1_, monoclonal isotype control, ab81032; Abcam) during the entire 2-week culture period. Matrix sGAG in the pellets was histologically stained with Safranin O, and chondrogenesis was analyzed according to a previously published histological pellet scoring system [32]. sGAG content was quantified using the Blyscan assay and normalized to double-stranded DNA content (Picogreen; Life Technologies). Gene expression in the pellets was evaluated by quantitative RT-PCR (see later).

Data were obtained from three independent experiments, with each experiment using pooled CSPCs from four or five different donors (*n* = 12).

### RNA extraction and quantitative RT-PCR

RNA was isolated using the RNeasy Mini Kit (QIAGEN, Valencia, CA, USA) according to the manufacturer’s instructions. Quantitative RT-PCR was performed using SYBR Green PCR Master Mix (Life Technologies) with a StepOne Plus Realtime PCR system (Applied Biosystems, Thermo Fisher, Grand Island, NY, USA). The PCR cycling consisted of 45 cycles of amplification of the template DNA with primer annealing at 60 °C. The relative level of gene expression was calculated using the 2-delta delta Ct method. *18S rRNA, RPL13a*, and *GAPDH* were chosen as housekeeping genes, which in general yielded similar results. The primer sequences used in this study are listed in supplementary materials (Additional file 1: Table S1).

### Zymography

Zymography was performed in 10 % gelatin polyacrylamide gel (Novex; Life Technologies). Medium samples (10 μl each) were mixed with zymogram sample buffer (BioRad, Hercules, CA, USA) and subjected to SDS-PAGE. The gels were then equilibrated with renaturation buffer (BioRad) and incubated with development buffer (BioRad) overnight at 37 °C. Bands were visualized by staining gels with Simply Blue Safe Stain (Invitrogen, Themo Fisher, Grand Island, NY, USA). At least two replicates were carried out for each sample analyzed by zymography. The MMP2 and MMP9 bands were digitally imaged and semiquantified by Image J 1.45s (NIH, Bethesda, MD, USA).

### MMP activity assay

5-FAM-Pro-Cha-Gly-Nva-His-Ala-Dap(QXL™520)-NH_2_ (Anaspec, Fremont, CA, USA), the fluorogenic MMP substrate XI [33, 34], was added into medium samples at a final concentration of 8.33 ng/μl. Fluorescence measurements (Ex/Em = 485/520 nm) were taken every 5 minutes for 1 hour in a microplate reader to determine substrate cleavage kinetics. Enzyme activities based on reaction rates were normalized to the original tissue wet weight.

### ELISA

Medium samples from IL-1β-treated chondrocytes were collected as already described. Chondrocytes from five patients were pooled for the test, and NGF concentrations (pg/ml) in the medium samples were determined using a commercially available ELISA kit (R&D Systems). All assays were carried out in triplicate. Total amounts of NGF released (pg) were calculated and normalized to double-stranded DNA content (mg), and values are shown as mean ± standard error (SE).

### Immunoblotting

Proteins were extracted using RIPA buffer (Sigma) and protease inhibitor cocktail (Sigma), boiled in sample buffer (BioRad), and separated by SDS-PAGE using 6 % stacking gel and 12 % separating gel (cell sample: 50 μg/lane; medium sample: 20 μl/lane). PVDF (0.45 μm; Millipore, Billerica, MA, USA) blots were prepared and incubated at 4 °C overnight with the primary antibodies (1:1000; anti-MMP3, Abcam; anti-TIMP1, R&D; anti-GAPDH, Abcam), followed by enzyme-conjugated secondary antibodies (GE, Marlborough, MA, USA), and detection was carried out with the ECL Kit (Pierce, Thermo Fisher, Grand Island, NY, USA) and visualized using a FOTO/Analyst1 Fx CCD imaging system (Fotodyne, Hartland, WI, USA). Images were analyzed by NIH Image J 1.45s. Each blot was repeated at least in duplicate, and representative scans are presented.

### Statistical analysis

Analysis of results from at least three independent experiments was performed using SPSS 16.0 software(SPSS Inc. Chicago, IL, USA). Results are reported as mean ± SD, unless specified otherwise. Student’s *t* test was performed between two groups. For experiments with more than two groups and multiple time points, after testing for normal distribution and variance homogeneity, a two-way/split plot analysis of variance (ANOVA) and post-hoc pairwise comparison of mean values were carried out. Statistical significance was considered at *p* <0.05.

## Results

### NGFRs are expressed in OA cartilage

**Fig. 1:**
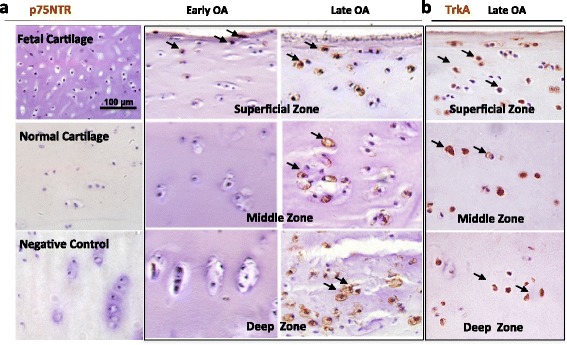
Immunodetection of NGF receptors in OA articular cartilage. **a** Immunostaining of the NGF low-affinity receptor, CD271 (or p75NTR), in fetal (*n* = 3, biological replicates), healthy (normal, *n* = 3, biological replicates), and OA (early and late stage, *n* = 5 each, biological replicates) hyaline cartilage from human femoral condyles, showing the superficial zone, middle zone, and deep zone. **b** Immunostaining of the NGF high-affinity receptor, TrkA, in OA (late stage) hyaline cartilage from human femoral condyles, showing the superficial zone, middle zone, and deep zone. CD271/p75NTR and TrkA presence is revealed by DAB staining (*brown*). *n* = 3–5 biological replicates. Negative control, without the use of primary antibody, is at the *bottom-left*. Bar = 100 μm. Arrows, positive signals of DAB staining. *OA* osteoarthritis, *p75NTR* low-affinity NGFR, *TrkA* high-affinity NGFR

Expression of the high-affinity NGFR, TrkA, was reported in adult articular cartilage, and reported to be higher in OA cartilage [23]. We examined the expression pattern of both TrkA and the low-affinity NGFR, p75NTR (also known as the stem cell marker CD271), in human knee articular cartilage sections by immunohistochemistry. CD271 staining was negative in fetal and normal adult human cartilage, but positive in the superficial zone of early OA articular cartilage (Fig. 1a). (Note: CD271/p75NTR-positive cells were seen in normal rabbit articular cartilage, suggesting species difference (see Additional file 1: Figure S1).) As OA progressed, cells were strongly positive for CD271 and TrkA appeared in all zones in late OA articular cartilage (Fig. 1a, b). The presence of both NGF and its high/low-affinity receptors in OA cartilage suggests that NGF signaling activity may be involved in OA pathogenesis.

### IL-1β regulates NGF/NGFR expression in chondrocytes

**Fig. 2:**
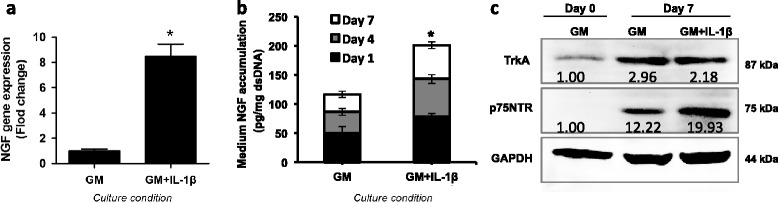
IL-1β stimulation of NGF/NGFR expression and release in human chondrocytes. Adult human chondrocytes in culture were treated with 100 pg/ml IL-1β in growth medium (*GM*) consisting of DMEM with 10 % FBS. **a** Higher *NGF* gene expression in response to treatment with IL-1β (GM versus GM + IL-1β; *p <0.05), analyzed by real-time RT-PCR on day 7 of treatment, with RPL13a as housekeeping gene control. **b** Higher NGF secretion in culture medium in response to IL-1β treatment (GM versus NGF; *p <0.05), measured by ELISA and expressed relative to double-stranded DNA content. **c** Higher NGFR expression in human chondrocytes in response to IL-1β analyzed by western blotting. Ten biological cell donors were analyzed. Experiments were carried out with three technical replicates using three pooled batches of samples, with each batch consisting of five randomly chosen samples. Images were digitally scanned and values are normalized to GAPDH and day 0 GM group, and expressed as mean ± SE. Representative images from one batch experiment are shown. *GAPDH* protein loading control, *IL* interleukin, *NGF* nerve growth factor, *p75NTR* low-affinity NGFR, *TrkA* high-affinity NGFR

We observed that NGF and its receptors, TrkA and p75NTR, were expressed in human articular cartilage-derived chondrocytes and their levels were regulated by the OA mediator, IL-1β. After culturing in 100 pg/ml IL-1β (level similar to that found in human OA synovial fluid) [35, 36], chondrocytes isolated from macroscopically asymptomatic articular cartilage in total knee arthroplasty samples showed higher levels of NGF gene expression (Fig. 2a). Higher levels of NGF protein were also detected by ELISA in the culture medium of IL-1β-treated cells (Fig. 2b), suggesting that NGF expression could be one of the cellular responses of chondrocytes to the proinflammatory effects of IL-1β.

Both NGFRs, the high-affinity NGFR, TrkA, and the low-affinity NGFR, p75NTR, were detected in cultured chondrocytes by immunoblotting (Fig. 2c), suggesting that chondrocytes have the potential to respond to NGF. In addition, the level of p75NTR was increased in the IL-1β treatment chondrocytes.

### CSPCs migrate towards NGF

**Fig. 3:**
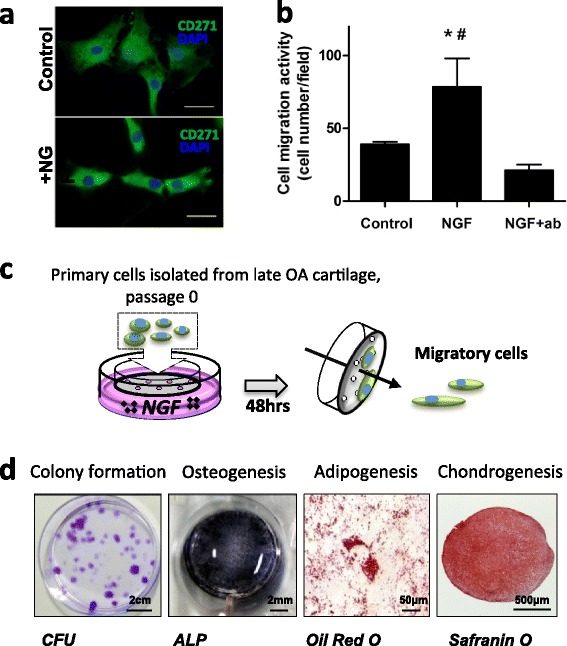
Response of CSPCs to NGF. **a** Change in morphology of CD271^+^ cells upon NGF treatment (10 ng/ml, 24 hours): CD271 (*green*), nuclei (*blue*). Bar = 50 μm. **b** Cells isolated from late OA articular cartilage are chemotactic for NGF (10 ng/ml). *p* <0.05, *versus control group, #versus NGF + ab group (*n* = 3, mean ± SD). **c** Schematic chart of isolation NGF chemotactic cells with Transwell. **d** Characteristics of NGF chemotactic cells: clonogenicity and differentiation multipotency. bar = 2 cm; osteogenesis, alkaline phosphatase (*ALP*), bar = 2 mm; adipogenesis, lipid droplet, Oil-red O; bar = 50 μm; chondrogenesis of pellet culture, GAG staining with Safranin O, bar = 500 μm. *ab* NGF neutralizing antibody, *CFU* crystal violet, *NGF* nerve growth factor, *OA* osteoarthritis

The surface marker CD271 was present at abundant levels in late OA cartilage. Late OA cartilage-derived primary cells were therefore used to examine the effects of NGF treatment. Upon treatment with 10 ng NGF/ml for 24 hours, we observed a change in the morphology of CD271^+^ cells from a well-spread shape to a more compact phenotype with defined cell borders (Fig. 3a).

As migratory activity is characteristic of CSPCs [29, 30], we analyzed cell migration in a Transwell setup. NGF recruited more cells migrating through the membrane than culture medium alone (Fig. 3b). Specifically, this promigration effect was inhibited by the addition of NGF neutralizing antibody. To confirm the CSPC characteristics of the migratory cells, cells collected from the bottom of the insert in the Transwell setup (Fig. 3c) were analyzed for their colony-formation ability, differentiation multipotency (Fig. 3d), and the presence of mesenchymal stem cell (MSC) -associated surface markers (see Additional file 1: Figure S2). These cells showed a colony-forming efficiency of 38.6 ± 14.8 % at passage 4; they also positively responded to osteogenic, adipogenic, and chondrogenic induction (Fig. 3d). Flow cytometry showed that these cells were positive for MSC surface markers CD90, CD73, CD105, CD166, CD44, and CD29, and negative for the hematopoietic stem cell markers CD34 and CD45, indicating a similar epitope profile between these cells and adult MSCs (see Additional file 1: Figure S2).

### CSPCs lost the CD271^+^ phenotype during in vitro culture

**Fig. 4:**
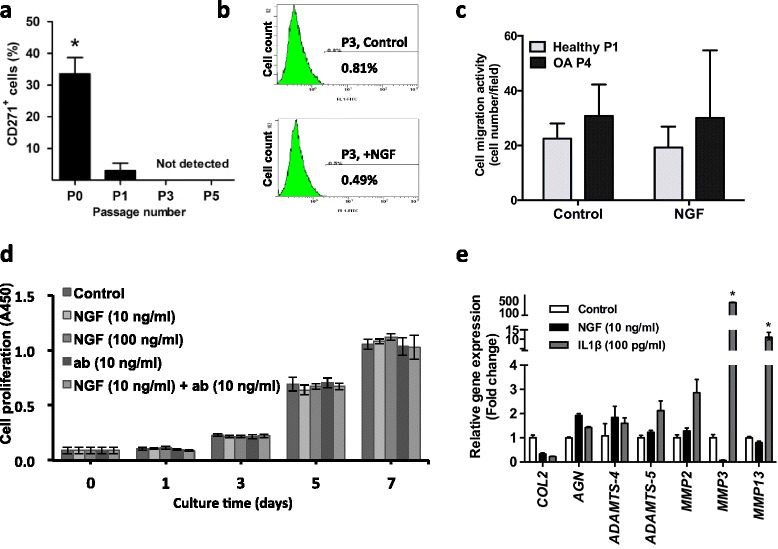
CD271^+^ cartilage-derived cells: loss of CD271^+^ phenotype and response to NGF during in vitro culture*.* **a** Abundance of CD271^+^ cells in the cartilage-derived cells determined by flow cytometry. Passages in culture: P0, 33.60 ± 5.09 %; P1, 3.00 ± 2.33 %; P3 and P5, signal undetectable (P0 versus P1: *p <0.05; *n* = 5, biological replicates; mean ± SD). **b** CD271^+^ cells (%) at P3, after treatment with 10 ng/ml NGF for 24 hours. Representative sample is shown (*n* = 3). **c** Early-passage cells isolated from healthy cartilage (passage 1 (*P1*); *n* = 3, biological replicates, mean ± SD) and late passage from OA articular cartilage (passage 4 (*P4*); *n* = 5, biological replicates, mean ± SD) are not chemotactic for NGF (10 ng/ml), *p* >0.05. **d** CSPC proliferation (*n* = 4). **e** Gene expression profiles of *COL2, AGN*, *MMP2/3/13*, and *ADAMTS-4/5*, in CSPCs before and after NGF (10 ng/ml, 48 hours) and IL-1β (100 pg/ml, 48 hours) treatment. IL-1β versus control: *p <0.05, *n* = 5, biological replicates, mean ± SD, values normalized to RPL13a. *ab* NGF neutralizing antibody, *ADAMTS* a disintegrin-like and metalloproteinase with thrombospondin motifs, *IL* interleukin, *MMP* matrix metalloproteinase, *NGF* nerve growth factor, *OA* osteoarthritis

The surface marker CD271 remained present (34 % positive) in primary cells isolated from late OA cartilage, and then rapidly disappeared after their culture passaging, as demonstrated by immunofluorescence and flow cytometry analyses (Fig. 4a). NGF treatment was not able to recover the CD271^+^ phenotype in cells that had been cultured in monolayer for three passages (Fig. 4b). Interestingly, healthy cartilage-derived primary chondrocytes or late passage OA-derived chondrocytes lost their chemotaxis towards NGF (Fig. 4c).

The effects of NGF on CSPCs were further examined to exclude the possibility that NGF and NGF neutralizing antibody increased cell numbers to cause false positives in the Transwell test. Unlike the effect observed on cell migration (Fig. 3b), neither treatment with NGF (10 and 100 ng/ml) nor with NGF antibody (10 ng/ml) affected cell proliferation (Fig. 4d).

We further tested the effects of NGF and IL-1β on the in vitro cultured CSPCs. No changes were seen in the expression levels of the cartilage matrix genes, *COL2* and *AGN*, and the OA-associated matrix degeneration genes, *MMP2/3/13* and *ADAMTS-4/5*, under NGF treatment, but treatment with IL-1β significantly increased *MMPs* (Fig. 4e). Thus, NGF did not appear to act on chondrocytes in a manner similar to the more generic proinflammatory cytokines, such as IL-1β [37–40].

To further study the influence of NGF in OA cartilage, it is necessary to maintain the NGFR^+^ phenotype of CSPCs. OA cartilage explants were therefore used as a model to study influences on tissue degeneration.

### NGF exposure increases matrix degradation in OA human cartilage explants

**Fig. 5:**
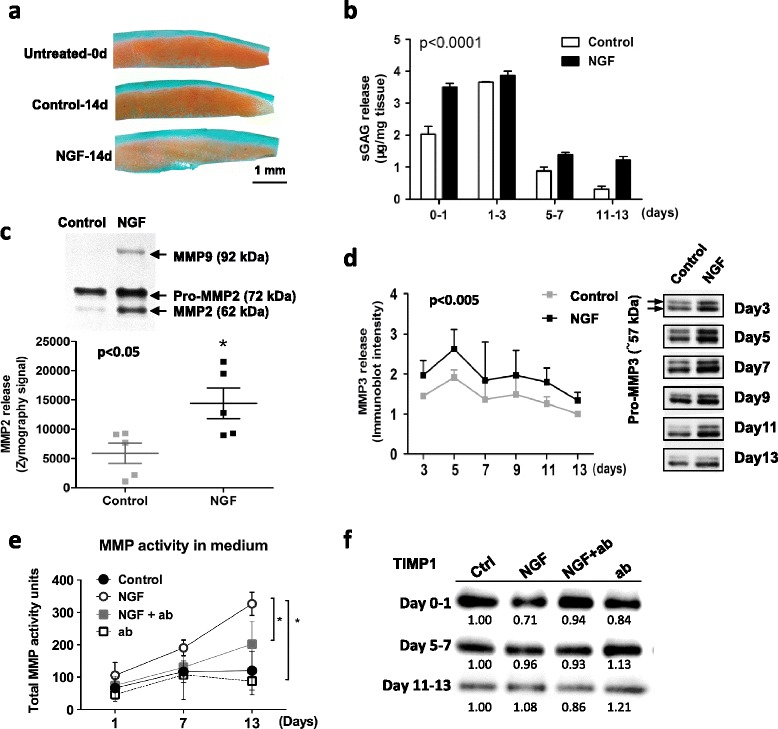
NGF treatment results in matrix degeneration in OA cartilage. Cartilage explants from OA tissue were cultured in serum-free medium (control), or treated with NGF for 14 days, and then the explants and culture medium were analyzed separately. **a** Safranin O staining of cartilage explants isolated from a uniform region before and after 14 days of culture, with or without 10 ng/ml NGF. **b** Medium sGAG release, normalized to tissue wet weight (mg) (*n* = 4, biological replicates). Representative samples are presented: values are mean ± SE; NGF versus control, two-way ANOVA, *p* <0.0001). **c** MMP zymography analysis of medium MMP2 and MMP9 activity (*n* = 5; representative images shown). Zymography analysis of MMP2 release in medium at day 1 (*n* = 5 biological replicates; *p <0.05), semiquantified based on band intensity signal. **d** Western blot analysis of medium MMP3 release during culture; semiquantified based on band intensity (*n* = 3 biological replicates; values are mean ± SD; NGF versus control, two-way ANOVA, *p* <0.005; blots from same patient sample). **e** Medium MMP activity. Medium samples from day 1, days 5–7, and days 11–13 were assayed (*n* = 4, biological replicates; NGF versus control group, and NGF versus NGF + ab group at day 13; *p <0.05, values are mean ± SD). **f** Western blot analysis of medium TIMP1 release at days 1, 7, and 13 (*n* = 4, biological replicates; representative results shown). *ab* NGF neutralizing antibody (10 ng/ml), *MMP* matrix metalloproteinase, *NGF* nerve growth factor, *sGAG* sulfated glycosaminoglycan, *TIMP1* tissue inhibitor of matrix metalloprotease-1

To investigate the effects of NGF on OA cartilage, human cartilage tissue explants were harvested from early OA joints and cultured in serum-free medium containing 10 ng/ml NGF for 14 days. The culture medium was collected at 24 hours after treatment, and every 48 hours thereafter. Histological examination of tissue sections with Safranin O staining (Fig. 5a) and colorimetric quantitation of sGAG content in the culture medium (Fig. 5b) showed that NGF treatment increased sGAG loss and release from cartilage explants.

Because of their known involvement in cartilage matrix degradation, production of MMPs in the cultured explants was analyzed. NGF treatment resulted in higher MMP levels in OA cartilage explants (Fig. 5e). Specifically, we analyzed the explant culture medium for MMP2 and MMP9 activity by means of zymography (Fig. 5c). MMP9 activity was turned on rapidly by NGF on day 1 (Fig. 5c), and remained elevated for the remainder of the culture period. Likewise, MMP2 activity was significantly increased upon NGF treatment (*p* = 0.038, *n* = 5) (Fig. 5c). Immunoblotting results also showed that more MMP3 was released into the culture medium from the NGF-treated explants (*p* = 0.040, *n* = 3) (Fig. 5d). Interestingly, NGF treatment appeared to increase matrix degeneration only in OA cartilage tissue; in isolated CSPCs, no changes were seen in the expression levels of cartilage matrix genes and OA-associated matrix degeneration genes (Fig. 4e).

To further assess the potential effects of NGF signaling in matrix metabolism, OA cartilage explants were treated with 10 ng/ml NGF and/or NGF neutralizing antibody (Fig. 5e). We observed that perturbing NGF signaling by adding NGF or NGF neutralizing antibody affected matrix degeneration. NGF increased MMP activity, and NGF antibody treatment blocked MMP activity (Fig. 5e). Interestingly, NGF signaling also affected the level of tissue inhibitor of matrix metalloprotease-1 (TIMP-1) in OA cartilage. NGF treatment reduced TIMP-1 on day 1, while the addition of NGF antibody increased TIMP-1 release on days 5–7 (Fig. 5f).

Taken together, these results strongly suggest that NGF plays a role in cartilage degeneration. It is possible that IL-1β activates the NGF/NGFR(s), and OA cartilage responds to NGF via CSPCs. That CSPCs are activated via IL-1β/NGF signaling represents a potentially new cell target in OA pathogenesis.

### NGF effects on CSPC matrix production and remodeling

**Fig. 6:**
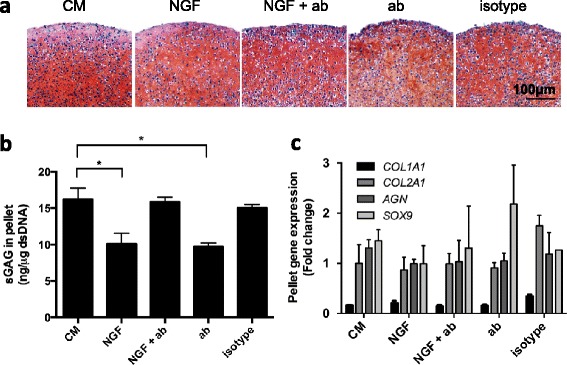
NGF signaling is partially involved in matrix remodeling in chondrogenic CSPCs. High-density pellets were formed using 2 × 10^5^ CSPCs, cultured in chondrogenesis medium (*CM*) for the first week, and then treated with 10 ng/ml NGF, NGF and NGF neutralizing antibody (*NGF + ab*), 10 ng/ml NGF neutralizing antibody (*ab*), or 10 ng/ml NGF neutralizing antibody matched IgG_1_ (*isotype*). Pooled CSPCs were used for the pellet study, derived from 14 patients and randomly distributed in five independent trials. Representative data presented are from five technical replicates for each group. **a** Safranin O staining of CSPC pellets at day 14. Bar = 100 μm. **b** sGAG quantification in pellets, normalized to double-stranded DNA. *p <0.05, versus CM group, values are mean ± SD. **c** Gene expression of *COL1A1*, *COL2A1*, *AGN*, and *SOX9* in CSPC pellets analyzed by quantitative RT-PCR, with RPL13a as housekeeping gene. Values are mean ± SD. *NGF* nerve growth factor, *sGAG* sulfated glycosaminoglycan

To investigate the functional role of NGF in cartilage matrix remodeling, OA cartilage-derived CSPCs were cultured as 3D pellets in chondrogenic medium and treated with NGF in combination with the NGF neutralizing antibody. The cultured cell pellets were harvested on day 14 and evaluated histologically by Safranin O staining, and scored on the basis of staining intensity, cell-to-cell distance, and cell morphology, according to a pellet scoring system [32] (Fig. 6a). With NGF and NGF blocking antibody treatment for a time span from day 7 to day 14, the matrix genes, *COL1A1*, *COL2A1*, *AGN*, and *SOX9*, did not significantly change at day 14 (Fig. 6c), and the histological pattern also did not change in the CSPC pellets. However, treatment with NGF or with NGF antibody alone reduced sGAG accumulation in the pellets (Fig. 6b). Perturbing NGF signaling by treatment with NGF or NGF neutralizing antibody therefore did not inhibit matrix production, but there appeared to be reduced matrix accumulation.

## Discussion

**Fig. 7:**
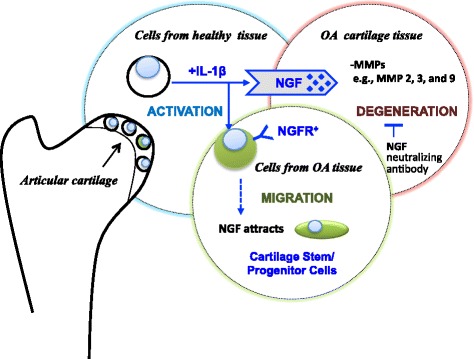
Schematic of NGF role and IL-1β involvement in OA. See text for details. *IL* interleukin, *MMP* matrix metalloproteinase, *NGF* nerve growth factor, *NGFR* nerve growth factor receptor, *OA* osteoarthritis

Increased IL-1β and NGF levels in the synovial fluid are symptoms of arthritis [23, 41]. It is well recognized that IL-1β is involved in cartilage degeneration and NGF mediates neuropathic pain in the disease state of OA [14], but the role of NGF in cartilage degeneration is unclear. In the present study, we have explored the role of IL-1β/NGF signaling in OA articular cartilage. Expression of NGF and NGFR was elevated in healthy human chondrocytes, in response to the proinflammatory OA inducer, IL-1β (Fig. 2). NGF treatment increased OA cartilage matrix degradation (Fig. 5), with NGF/NGFR action targeting CSPCs, illustrated by NGF induction of chemotactic CSPC migration (Fig. 3). Our findings also showed that NGF signaling affected cartilage tissue integrity and related cell activities (Fig. 5), as well as matrix accumulation in chondrogenic cultures of CSPCs (Fig. 6). Taken together, these findings strongly suggest the functional involvement of IL-1β/NGF signaling in OA pathogenesis, acting through CSPCs (Fig. 7).

CSPCs were initially found and associated with tissue injury [30] or degenerative and inflammatory conditions as in OA, and migratory activity has been recognized as a characteristic behavior of CSPCs [29, 30]. We found here that cells isolated from late OA cartilage are chemotactic for NGF. Interestingly, the low-affinity NGFR, also known as CD271, an early stem cell marker, is highly expressed in late OA cartilage (Fig. 1). We also observed that only cells from late OA articular cartilage, but not cells from normal cartilage, are chemotactic for NGF (Fig. 4c). We therefore speculate that CD271^+^ cells are the NGF responsive CSPCs in OA cartilage. We found that the CD271^+^ cells are unable to maintain their phenotype after isolation and passage in vitro (Fig. 4a). Interestingly, we also observed species variation, as rabbit articular cartilage displays a different distribution of CD271^+^ cells, compared with human (see Additional file 1: Figure S1). Further studies in human and other appropriate animal models are needed and under development to verify that the CD271^+^ cells represent the sole or primary NGF chemotactic migratory cell subpopulation.

We observed that IL-1β acts upstream of NGF in OA-related NGF/NGFR signaling (Fig. 2). Specifically, treatment of chondrocytes with IL-1β increases NGF mRNA and protein levels, and both high-affinity and low-affinity NGFRs—TrkA [22] and p75NTR/CD271, respectively—are positively immunostained in OA articular cartilage [23]. Therefore, IL-1β could be involved in the activation of the NGF responsive cells in healthy cartilage (Fig. 7). We also found that NGF/NGFR is involved in OA cartilage degeneration, as NGF treatment increases matrix degradation in OA cartilage explants, indicated by increased GAG release and MMP activity, particularly MMP2, MMP3, and MMP9. Taken together, these findings strongly support the functional involvement of IL-1β/NGF signaling in the pathogenesis of OA. However, it is still unclear whether the CSPCs are quiescent stem/progenitor cells that are activated by IL-1β, or whether the healthy chondrocytes after treatment with IL-1β convert to NGF responsive cells. Additional study is clearly needed.

Clinical studies with NGF neutralizing antibody have demonstrated its beneficial effects in reducing pain levels in the treatment of OA [15]. In animal studies, blocking NGF and NGFR-mediated signaling through the use of neutralizing antibody to NGF [19] or to the NGFR, TrkA [20, 42], could reduce OA pain [20, 21]; however, the effect on joint cartilage health is unknown. We therefore examined the effects of inhibition of NGF on the biological activities of articular cartilage and cartilage-derived cells. We observed that NGF-mediated CSPC migration is blocked by treatment with NGF neutralizing antibody (Fig. 3b). In addition, MMP activity is also partially suppressed by treatment with NGF neutralizing antibody in cultured cartilage explants (Fig. 5e). These results suggest that blocking NGF may affect joint cartilage via cell-mediated mechanism, in addition to the reported pain relief [15]. Interestingly, the NGF effect on MMPs is only observed in OA cartilage explants, but not in the cultured chondrocytes (e.g., lack of stimulation of MMP13 and ADAMTS-5 shown in Fig. 4e), suggesting the importance of the diseased tissue environment. Previous study of relevance is an animal experiment showing that NGF-induced weight-bearing asymmetry in non-OA rats was normalized to that of saline-injected controls by 1 week after NGF antibody injection, whereas pain behavior remained augmented in NGF-injected OA rats [21]. Taken together, these findings strongly suggest that while NGF may not be the initiator of OA, it is likely to contribute to OA pathogenesis, not only via neuropathic pain but also via pathological changes in cartilage structure by targeting the CSPCs [43].

To examine the functional role of NGF signaling in CSPCs, we applied an in vitro 3D, chondrogenic CSPC pellet culture model. Our results showed that perturbing NGF signaling with NGF (10 ng/ml) and NGF neutralizing antibody resulted in less cartilage ECM accumulation (Fig. 6b). However, the general histological structure and expression of matrix genes was not affected (Fig. 6c). These findings suggest that NGF may partially function in modulating the CSPC matrix assembly process. It is interesting that NGF neutralizing antibody alone lowers matrix accumulation, suggesting possible biological activity of the endogenous traces amount of NGF (~pg/μg double-stranded DNA, see Fig. 2b). Further study is therefore needed to clarify this role.

We also tested whether blocking NGF may affect other activities of joint cartilage in addition to the reported pain relief [15]. Interestingly, treatment with NGF neutralizing antibody reduced MMP activity and maintained TIMP1 production in OA cartilage explants (Fig. 5f), suggesting the possibility that blockade of NGF could reduce matrix loss in OA cartilage. Because of the species variation of NGFR distribution (Additional file 1: Figure S1) and the challenge in maintaining NGFR expression in vitro (Fig. 4), an appropriate animal model is needed in the future to study the long-term in vivo effects of the NGF signaling blockades.

## Conclusions

The results reported here strongly suggest that NGF signaling is a contributing factor in articular cartilage degeneration in OA, which probably targets a specific subpopulation of progenitor cells, the CSPCs. More in-depth knowledge of the molecular and cellular mechanism of IL-1β/NGF signaling will be crucial in exploring its potential utility as a therapeutic target for OA.

## Additional file

Additional file 1:
**Figure S1 showing immunodetection of CD271/p75NTR in normal rabbit cartilage, Figure S2 showing characterization of the cell surface epitope profile of migratory cells isolated from human OA articular cartilage, Figure S3 showing the gene expression profile of human OA cartilage explants after NGF (10 ng/ml) treatment for 14 days, and Table S1 presenting primer sequences for real-time RT-PCR.** (DOCX 1277 kb)

## Abbreviations

ADAMTS: A disintegrin-like and metalloproteinase with thrombospondin motifs
ALP: Alkaline phosphatase
ANOVA: Analysis of variance
CDC: Centers for Disease Control and Prevention
CSPC: Cartilage stem/progenitor cell
3D: Three-dimensional
DAPI: 4′,6-Diamidino-2-phenylindole
DMEM: Dulbecco’s Modified Eagle’s medium
ECM: Extracellular matrix
ELISA: Enzyme-linked immunosorbent assay
FBS: Fetal bovine serum
FITC: Fluorescein isothiocyanate
IL: Interleukin
MMP: Matrix metalloproteinase
NGF: Nerve growth factor
NGFR: Nerve growth factor receptor
NSAID: Nonsteroidal anti-inflammatory drug
OA: Osteoarthritis
p75NTR: p75 neurotrophin receptor, CD271, NGF low-affinity receptor
PE: Phycoerythrin
PG: Proteoglycan
SD: Standard deviation
SE: Standard error
sGAG: Sulfated glycosaminoglycan
TIMP-1: Tissue inhibitor of matrix metalloprotease-1
TNFα: Tumor necrosis factor alpha
TrkA: Tropomyosin receptor kinase A, NGF high-affinity receptor
