# Supplementary material for: Cartilage stem/progenitor cells are activated in osteoarthritis via interleukin-1β/nerve growth factor signaling
Source: Arthritis Res Ther. 2015 Nov 17;17:327. doi: 10.1186/s13075-015-0840-x (PMC4650403; doi:10.1186/s13075-015-0840-x)
Supplement: Additional file 1: — Figure S1 showing immunodetection of CD271/p75NTR in normal rabbit cartilage, Figure S2 showing characterization of the cell surface epitope profile of migratory cells isolated from human OA articular cartilage, Figure S3 showing the gene expression profile of human OA cartilage explants after NGF (10 ng/ml) treatment for 14 days, and Table S1 presenting primer sequences for real-time RT-PCR. (DOCX 1277 kb) [file 13075_2015_840_MOESM1_ESM.docx]

**Additional file 1**


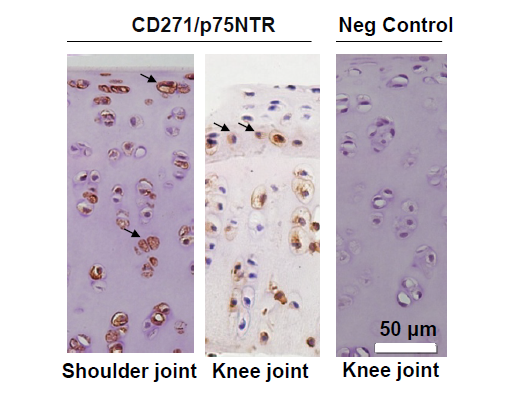


**Fig. S1.** Immunodetection of CD271/p75NTR in normal rabbit cartilage

Strong CD271 immunostaining was seen in normal shoulder and knee articular cartilage of adult New Zealand White rabbit (age: 3-4 months). Neg Control, negative control without primary antibody. Bar = 50 μm.

**
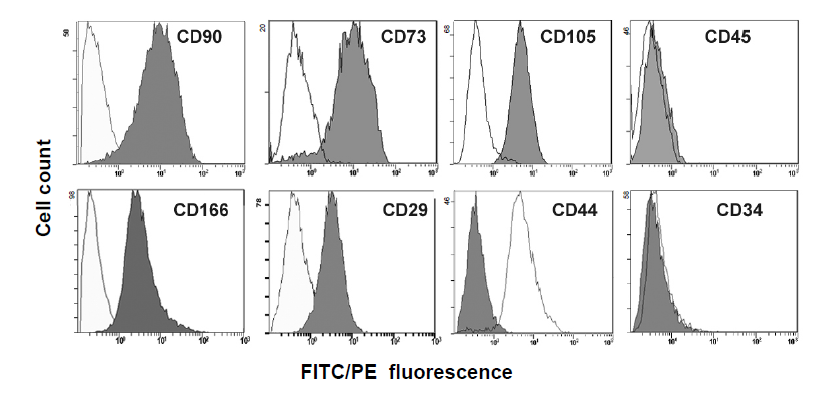
**

**Fig. S2.** Characterization of cell surface epitope profile of migratory cells isolated from human OA articular cartilage.

Flow cytometric analysis of stem cell-relevant surface markers revealed the presence of CD90, CD73, CD105, CD166, CD29, and CD44, and absence of CD34, and CD45.

**
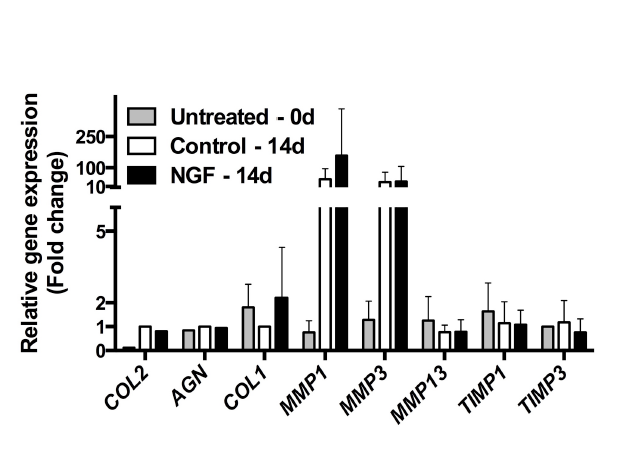
**

**Figure S3.** Human OA cartilage explants gene expression profile after NGF (10 ng/ml) treatment for 14 days.

Cartilage explants from OA tissue were cultured in serum free medium (control), or treated with NGF for 14 days, and then the gene expression of the explants were analyzed separately. (N=10, biological replicates; Control *versus* NGF, no significant difference; values are mean ± S.D).

**Table S1.** Primer sequences for real-time RT-PCR

| Genes | NCBI  Gene ID | Primer sequences (5’-3’) | | Product size (bp) |
| --- | --- | --- | --- | --- |
| _______________ | ______________ | _____________________________________________________________________ | | __________ |
| COL1A1 | 1277 | Forward | CGATGGATTCCAGTTCGAGTAT | 248 |
|  |  | Reverse | CATCGACAGTGACGCTGTAGG |  |
| COL2A1 | 1280 | Forward | GGATGGCTGCACGAAACATACCGG | 157 |
|  |  | Reverse | CAAGAAGCAGACCGGCCCTATG |  |
| SOX9 | 6662 | Forward | AGCGAACGCACATCAAGAC | 84 |
|  |  | Reverse | CTGTAGGCGATCTGTTGGGG |  |
| AGN | 176 | Forward | AGTCACACCTGAGCAGCATC | 147 |
|  |  | Reverse | AGTTCTCAAATTGCATGGGGTGTC |  |
| ADAMTS-5 | 11096 | Forward | ATCACCCAATGCCAAGG | 246 |
|  |  | Reverse | AGCAGAGTAGGAGACAAC |  |
| MMP13 | 4322 | Forward | ATGCAGTCTTTCTTCGGCTTAG | 269 |
|  |  | Reverse | ATGCCATCGTGAAGTCTGGT |  |
| RPL13a | 23521 | Forward | CATAGGAAGCTGGGAGCAAG | 157 |
|  |  | Reverse | GCCCTCCAATCAGTCTTCTG |  |
| RNA18S5  (18S) | 100008588 | Forward | GTAACCCGTTGAACCCCATT | 151 |
|  |  | Reverse | CCATCCAATCGGTAGTAGCG |  |
| NGF | 4803 | Forward | CCAGTGGTCGTGCAGTCCAAG | 217 |
|  |  | Reverse | TGTCCTGCAGGGACATTGCTCT |  |
| IL-1β | 3553 | Forward | TTACAGTGGCAATGAGGATGAC | 134 |
|  |  | Reverse | TGCTGTAGTGGTGGTCGGAGA |  |
| ADAMTS-4 | 9507 | Forward | CTGGCACCTACCTGACTGG | **118** |
|  |  | Reverse | GTAACACGCCTAACAGGGCT |  |
| MMP3 | 4314 | Forward | AGTCTTCCAATCCTACTGTTGCT | **226** |
|  |  | Reverse | TCCCCGTCACCTCCAATCC |  |
| TIMP1 | 7076 | Forward | CTTCTGCAATTCCGACCTCGT | **79** |
|  |  | Reverse | ACGCTGGTATAAGGTGGTCTG |  |
| TIMP3 | 7078 | Forward | CATGTGCAGTACATCCATACGG | **100** |
|  |  | Reverse | CATCATAGACGCGACCTGTCA |  |
